# Evaluating PHA Productivity of Bioengineered *Rhodosprillum rubrum*

**DOI:** 10.1371/journal.pone.0096621

**Published:** 2014-05-19

**Authors:** Huanan Jin, Basil J. Nikolau

**Affiliations:** 1 Department of Biochemistry, Biophysics and Molecular Biology, Iowa State University, Ames, Iowa, United States of America; 2 Center for Metabolic Biology, Iowa State University, Ames, Iowa, United States of America; 3 Center for Biorenewable Chemicals, Iowa State University, Ames, Iowa, United States of America; University Paris South, France

## Abstract

This study explored the potential of using *Rhodosprillum rubrum* as the biological vehicle to convert chemically simple carbon precursors to a value-added bio-based product, the biopolymer PHA. *R. rubrum* strains were bioengineered to overexpress individually or in various combinations, six PHA biosynthetic genes (*phaC1*, *phaA*, *phaB*, *phaC2*, *phaC3, and phaJ*), and the resulting nine over-expressing strains were evaluated to assess the effect on PHA content, and the effect on growth. These experiments were designed to genetically evaluate: 1) the role of each apparently redundant PHA polymerase in determining PHA productivity; 2) identify the key gene(s) within the *pha* biosynthetic operon that determines PHA productivity; and 3) the role of *phaJ* to support PHA productivity. The result of overexpressing each PHA polymerase-encoding gene indicates that *phaC1* and *phaC2* are significant contributors to PHA productivity, whereas *phaC3* has little effect. Similarly, over-expressing individually or in combination the three PHA biosynthesis genes located in the *pha* operon indicates that *phaB* is the key determinant of PHA productivity. Finally, analogous experiments indicate that *phaJ* does not contribute significantly to PHA productivity. These bioengineering strains achieved PHA productivity of up to 30% of dry biomass, which is approximately 2.5-fold higher than the non-engineered control strain, indicating the feasibility of using this approach to produce value added bio-based products.

## Introduction

Since the industrial revolution humanity has increasingly become dependent on fossil carbon as a source of energy and chemicals. Most recently a number of factors have coalesced to provide the impetus to explore the use of biomass as an alternative, renewable source of carbon [1], [2]. The recalcitrant nature of biomass, resisting degradation to useful intermediates that can be used as feedstocks in either chemical or biological conversion processes is impeding this development. One possible route through this multifaceted conversion process is to thermochemically depolymerize the biomass and deconstruct it to chemically simple carbon precursors, which could be utilized by microbial fermentation to produce value-added products. Fast pyrolysis is a non-enzymatic, thermal depolymerization of biomass in the absence or low levels of oxygen, which can produce gaseous (syngas), liquid (bio-oil), or solid (biochar) energy-rich materials [3], [4]. Converting these materials to useful value-added products could be achieved via a fermentation-based method, but is hindered by the fact that these pyrolysis materials are relatively complex chemical mixtures, that also includes microbial growth inhibitors. The potential of using these deconstructed biomass materials as a carbon source for fermentation not only overcomes the expense associated with using a chemically more complex, recalcitrant carbon source (e.g., lignocelluloses), but also bypasses the high cost and poor yields of enzymatic hydrolysis of waste biomass into simple sugars that are suitable for fermentation.

*Rhodosprillum rubrum* is an attractive microbial fermentation organism for converting such deconstructed biomass-products to value-added biochemicals because it can utilize a variety of different carbon and energy source under anaerobic conditions [5], [6]. Because of its flexible capabilities to grow aerobically, anaerobically or as an autotroph, *R. rubrum* is particularly attractive for fermenting syngas feedstocks (particularly biomass-derived syngas), which are primarily a mixture of carbon monoxide, hydrogen, carbon dioxide and methane. In this study we explored the potential of using this Gram negative, photosynthetic purple non-sulfur bacterium for the production of valuable biochemicals from simple carbon feedstocks, using a transcription regulatory system that is inducible with carbon monoxide, which would therefore be applicable in developing a syngas fermentation platform.

The biochemicals we targeted for these bioengineering efforts are polyhydroxyalkanoates (PHAs), which are biodegradable polyester polymers that are deposited within inclusion bodies, and many microbes use them as a means of storing carbon and energy [7]–[9]. Because PHAs can be used as biodegradable plastics there has been a great deal of interest in generating a PHA production system based upon microbial fermentation [10]. *R. rubrum* has the potential of accumulating up to 50% of dry weight as PHA, using an optimal carbon source such as butyrate [11]. Recent studies have evaluated the technical and economic feasibility of using such a microbial platform for the conversion of biomass feedstocks to biofuels or biochemicals [12], [13], and one of the conclusions from these studies is that additional “metabolic engineering could be employed to increase yield and broaden the variety of available products” [14]. In this study we specifically targeted the bioengineering of *R. rubrum* PHA biosynthetic genes, and explored the effect of systematically overexpressing them on the production of PHA, when *R. rubrum* is grown in chemically simple carbon feedstocks in anaerobic conditions, and controlling the expression of the bioengineered genes with a carbon monoxide inducible promoter.

Previous studies of the *R. rubrum* genome have revealed the presence of three PHA polymerases that are designated *phaC1* (Rru_A0275), *phaC2* (Rru_A2413) and *phaC3* (Rru_A1816) [15]–[17], respectively, and one (*R*)-specific 2-enoyl-CoA hydratase (*phaJ* (Rru_A2964)) [18]. PhaJ converts *trans*-2-enoylacyl-CoA to (*R*)-3-hydroxyacyl-CoA, the substrate of PhaC [9]. The sequence identities shared between PhaC1, PhaC2 and PhaC3 are 14.3, 18.4 and 50.2%, respectively. The *phaC1* gene resides in the PHA biosynthetic operon that also includes the *phaA* (Rru_A0274) and *phaB* (Rru_A0273) genes. The products of this *phaC1-phaA-phaB* operon, are enzymes that sequentially catalyze condensation of two acetyl-CoA molecules to form acetoacetyl-CoA, the reduction of acetoacetyl-CoA to (*R*)-3-hydroxybutyryl-CoA, and the polymerization of 3-hydroxybutyrate to form the PHA, polyhydroxybutyrate [9]. In contrast, the *phaC2* and *phaC3* gene are located in other discrete locations in the genome.

## Materials and Methods

### Bacterial Strains, Plasmids and Culture Conditions

The strains of *R. rubrum* and *E. coli*, and the plasmids used in this study are listed in Table S1 in File S1. The Table S2 in File S1 lists DNA primers used in this study. *E. coli* was grown at 37°C in LB medium. *R. rubrum* was grown photo-heterotrophically under 5000 Lux light intensity, at 25°C in supplemented malate-ammonium medium (SMN medium; Table S3 in File S1) [19]. To assess PHA production, cells from a 0.5 ml aliquot of a normalized SMN culture (5 O.D.) were collected by centrifugation at 13,000×g for 2 min; the cells were washed once with nitrogen-limiting RRNCO medium (i.e., without ammonium chloride; Table S4 in File S1) [5] and re-suspended with 0.5 ml RRNCO medium and transferred to 50 ml RRNCO medium. The culture was incubated anaerobically with a carbon monoxide head-space, in 121 ml stoppered serum bottles at 25°C, under 5000 Lux light intensity, and shaken at 150 rpm. Four-milliliter aliquots of the cell cultures were harvested at 54, 66, 84, 98, 120, 146-hour cultivation for PHA content analysis. Cell density was determined using a Spectronic 20D+ spectrophotometer measuring absorbance at 680 nm (Thermo Fisher Scientific Inc., Waltham, MA). Doubling time (T_d_) was determined from the initial four time points of cultures. Antibiotics were used for selection of plasmids as follows: ampicillin 100 µg/ml (*E. coli*); kanamycin, 25 µg/ml (*R. rubrum*) or 50 µg/ml (*E. coli*). IPTG and X-gal were used at concentrations of 20 and 40 µg/ml, respectively.

### Gas Chromatographic Analysis of PHA

Cells were collected by centrifugation at 6000×g for 10 min and the pellet was washed once with 10 mM Tris-HCl buffer (pH 7.5) and lyophilized overnight. PHA content and compositions were determined as described by Brandl et al. [20]. For quantification purposes a known amount of hexadioic acid was added to the cell pellet as an internal control. The methyl esters were assayed by GC-MS with an Agilent 6890 GC equipped with DB-WAX column (30 m×0.25 mm ID, 0.5 µ m) interfaced to a 5973 mass spectrometer and an electron impact ionization detector (Agilent Technologies, Santa Clara, CA). The GC/MS data files were deconvoluted by NIST AMDIS software. The PHA content was calculated as the percent of cell dry weight (CDW). 3-hydroxybutyrate was used to construct a response standard curve, which was linear with respect to the cell mass used in the analysis. The detection limit of this method is 1 µg PHA/mg CDW. Data were analyzed using SAS software by two-way ANOVA. P-value less than 0.05 was used to evaluate the statistical significance difference.

### DNA Isolation and Manipulation

Genomic DNA was isolated from *R. rubrum* as described by Kerby et al. [19]. Plasmids were isolated from *E. coli* cells grown in LB medium by using QIAprep Spin Miniprep Kit (Qiagen Inc., Valencia, CA). Agarose gel electrophoresis and transformation of *E. coli* were carried out as described by Sambrook and Russell [21]. PCR products were cloned into pCR2.1-TOPO using a TOPO TA cloning kit (Invitrogen Corporation, Carlsbad, CA).

### Construction of Plasmids

**Figure 1:**
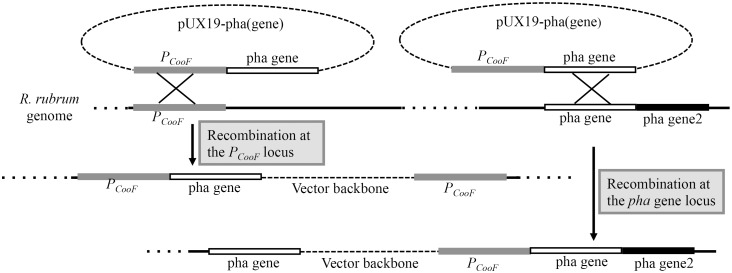
Recombination based integration of the CO-inducible *P_CooF_* promoter for bioengineering the overexpression of *pha* biosynthetic genes. Construction of the suicide plasmids pUX19-pha(gene) is described in the Material and Methods. Recombination at either the *P_CooF_* locus or the *pha* structural gene provides a mechanism for generating different strains listed in Table I that carry alleles for overexpressing the *pha* biosynthetic genes.

The plasmids for expressing protein in *E. coli* strain BL21-AI were constructed as recommended by Invitrogen Gateway technology. The plasmids pUX19-PT carrying different *pha* genes, and pUX19-PT-GUS, which were used to generate strains for over-expressing *pha* genes and the *gusA* gene, respectively, were constructed as follows. Using PCR, *XbaI* and *NdeI* sites were first introduced into the upstream and downstream regions of an 818-bp fragment that contains the promoter of the carbon monoxide inducible *R. rubrum CooFSCTJ* operon [22]. The *CooFSCTJ* promoter has been previously characterized as the 120-bp fragment that is immediately upstream of the translational start codon of the *CooF* gene. We targeted an 818-bp fragment immediately upstream of this translational start codon in order to encompass the promoter (*P_CooF_*) and ensure subsequent recombinational integration of bioengineered transgenes at the *CooF* locus (Fig. 1). The PCR product was purified and ligated into the TA cloning vector, pPCR2.1. The resulting vector was digested with *NdeI* and *SacI* and the *P_CooF_* promoter fragment was purified and cloned into pUX19 [23] digested with *XbaI* and *NdeI*, forming vector pUX19-P1. The 110-bp terminator of *CooFSCTJ* operon [15], and each PHA biosynthetic gene or the GUS gene were prepared and ligated sequentially into the vector pUX19-P1 with the same method, forming vectors pUX19-PT-(*pha* genes). In these constructs the ORFs of each PHA biosynthetic gene and the GUS gene were PCR amplified from the translational start and stop codons, using the DNA primers listed in Table S2 in File S1.

### Conjugation

The pUX19-PT-(*pha*-target gene) plasmids were mobilized from *E. coli* 17-1 into *R. rubrum* by conjugation conducted as described by Liang [24]. *R. rubrum* conjugants were selected on MN medium, supplemented with kanamycin. Repeated transfer of inoculum to kanamycin containing MN media, selecting a single colony after each transfer was used as a means of isolating *R. rubrum* conjugants. Two types of recombination outcomes were isolated (Fig. 1 and Table 1); one occurs at the *P_CooF_* promoter locus, and the second occurs at the structural *pha* gene that was included in the pUX19-PT-(*pha* genes) vector. In the second case, the expression of any other *pha* structural gene down-stream of the recombination site (i.e., *pha* gene 2, Figure 1) also became inducible via the CO-activation. All recombination integration sites were confirmed by PCR amplification, and DNA sequencing.

**Table 1:** Recombinant *R. rubrum* strains.

		Genetic location targeted for integration		Expected phenotypic modification
Gene	*R. rubrum* strains	*cooFSCTJ* Promoter	Structural Gene	
Vector control	oxControl	+		none
*phaC*	oxPHA(C1AB)		+	Entire *pha* operon induced
	oxPHA(C1)	+		*phaC1* only induced
*phaA*	oxPHA(AB)		+	Both *phaA* and *phaB* induced
	oxPHA(A)	+		*phaA* only induced
*phaB*	oxPHA(B)	+		*phaB* only induced
*phaC2*	oxPHA(C2)	+		*phaC2* only induced
*phaC3*	oxPHA(C3)	+		*phaC3* only induced
*phaJ*	oxPHA(J)	+		*phaJ* only induced
GUS	oxGUS	+		Expression of *GUS*; positive control

### GUS Activity Assay

Cells expressing the GUS reporter were collected and resupended in GUS assay buffer (1 mM EDTA, 50 mM NaHPO_4_−Na_2_PO_4_, 0.1% Triton X-100, pH 7.0) and were disrupted ultrasonically. GUS activity was determined as described previously [25]. One unit of GUS activity is defined as the formation of 1 mM *p*-nitrophenol formed per minute at 37°C.

### Protein Purification and Preparation of Antibodies

Recombinant His-tagged or GST-tagged fusion proteins were affinity-purified via their tags from extracts of *E. coli* BL21-AI strains harboring pDEST15-(*pha*-target gene) or pDEST17-(*pha*-target gene), grown in the presence of 0.2% L-arabinose. Recovered proteins were further purified via preparative scale SDS-PAGE. The recombinant protein bands was excised from gels, crushed in PBS solution and used to immunize mice and generate antibodies against each protein.

### SDS-PAGE and Western Immunoblot Analysis

Proteins were separated by SDS-PAGE in 12.5% polyacrylamide gels. Gels were loaded with aliquots containing of equal amounts of protein; protein concentrations were determined using the Bio-Rad (Hercules, CA) Dc Protein Assay Kit, using BSA to generate the standard curve. Proteins were electrophoretically transferred to a nitrocellulose membrane [21]. PHA biosynthetic proteins were immunologically detected with a combination of primary mice antibodies directed against the PHA proteins, and secondary HRP-conjugated anti-mouse IgG antibody (Bio-Rad) (Hercules, CA). The blot was developed with Amersham (Little Chalfont, Buckinghamshire, UK) ECL western blot detection reagents. For quantitative analysis, an Epson Perfection 2400 scanner (Shiojiri-shi, Nagano-ken, Japan) was used to scan the x-ray film. The signal intensities were quantified by ImageJ software (http://rsbweb.nih.gov/ij/index.html).

## Results

### Recombinant Strains Carrying CO-inducible-*Pha* Genes

**Figure 2:**
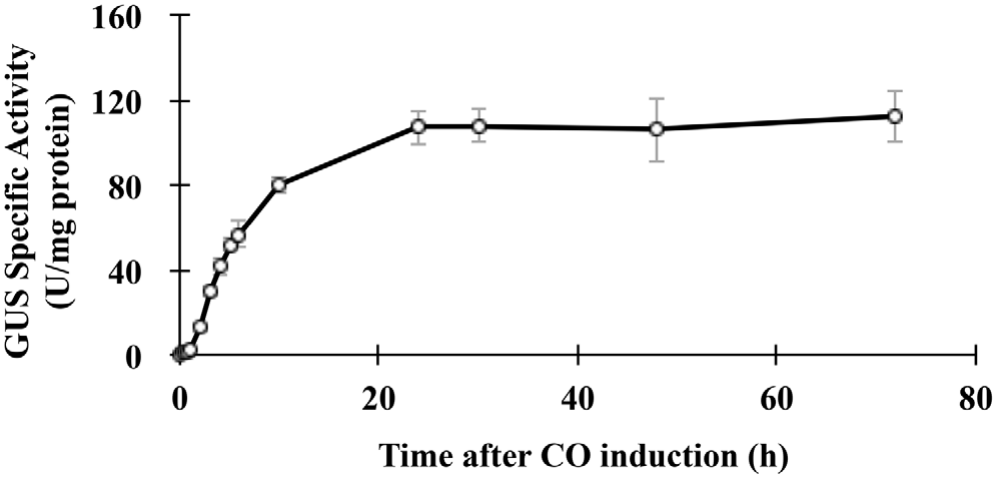
Induction of GUS-expression by carbon monoxide from an integrated P_CooF_−GUS-T_CooF_ locus. Data represent average from triplicate biological samples, and error bars indicate the standard error.

In this study we investigated the metabolic regulation of PHA biosynthesis by determining the *in vivo* effect of over-expressing the PHA biosynthetic genes on PHA productivity and culture-growth. In the resulting bioengineered *R. rubrum* strains the *PHA* biosynthetic genes were overexpressed individually or in combination using the strong, carbon monoxide inducible *cooFSCTJ* promoter [22]. To conduct these over-expression experiments we initially tested the efficacy of the carbon monoxide inducible system, by constructing the P*_CooF_*: GUS vector and generated a strain to evaluate GUS expression. The results establish that *cooFSCTJ* promoter activity (determined as GUS activity) is undetectable in the absence of carbon monoxide, and reaches maximum activity (>400-fold induction) within 24-h of exposure to carbon monoxide, reaching 50% of the maximum within the initial 5-hours (Fig. 2).

### Metabolic Engineering of *R. rubrum* by Over-expressing PHA Biosynthetic Genes

Bioengineered strains that over-express select *PHA* biosynthetic genes were constructed by introducing into *R. rubrum* each P*_CooF_* :*PHA* construct on a PUX19-based plasmid, and selecting for resistance to the antibiotic carried by that base-plasmid (Kan^R^). Because this plasmid cannot replicate in this host, two types of homologous recombination events will confer Kan^R^ strains, recombination at the *CooF* promoter locus or recombination at the *PHA* structural gene locus. For example, conducting such an experiment with the vector pUX19-*P_CooF_: phaC1* can result in: 1) recombination at the *CooF* promoter locus, generating a strain that has the *phaC1* gene under the control of *CooF* promoter and only this new, recombinant *phaC1* locus can be induced by carbon monoxide; or 2) recombination at the endogenous *phaC1* locus (Rru_A0275), generating a recombinant locus that is composed of a *CooF* promoter-driven transcription unit encoding *phaC1*, and the downstream located PHA structural genes within that operon (*phaA* and *phaB*). This recombinant locus would therefore become inducible with carbon monoxide. Using this strategy, ten different strains were constructed (Table 1). In Table 1, and in subsequent text herein we use the prefix “oxPHA” as the shorthand nomenclature to indicate which *PHA* biosynthetic genes have been overexpressed in the resulting recombinant strains.

**Figure 3:**
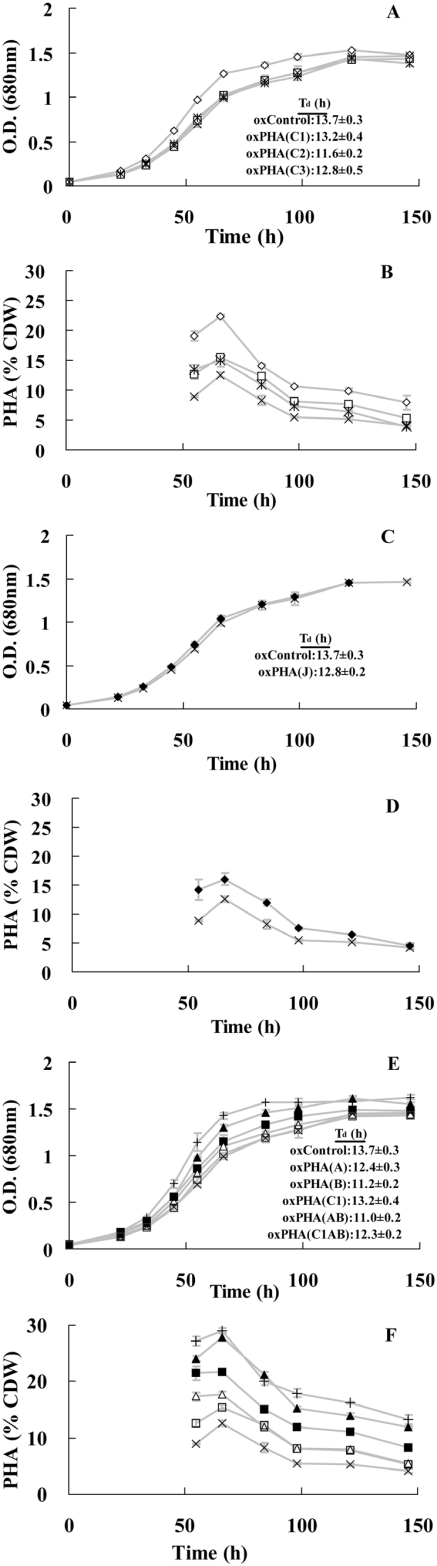
Growth curves (A, C and E, with doubling time indicated) and PHA content (B, D and F) of *R. rubrum* strains. *R. rubrum* strains were: oxControl (**×**), oxPHA(C1) (**□**), oxPHA(C2) (**◊**), oxPHA(C3) (*), oxPHA(A) (**△**), oxPHA(B) (**▴**),oxPHA(AB) (**+**), oxPHA(C1AB) (**▪**), oxPHA(J) (**♦**). Data represent average from triplicate biological samples, and error bars indicate the standard error.

**Figure 4:**
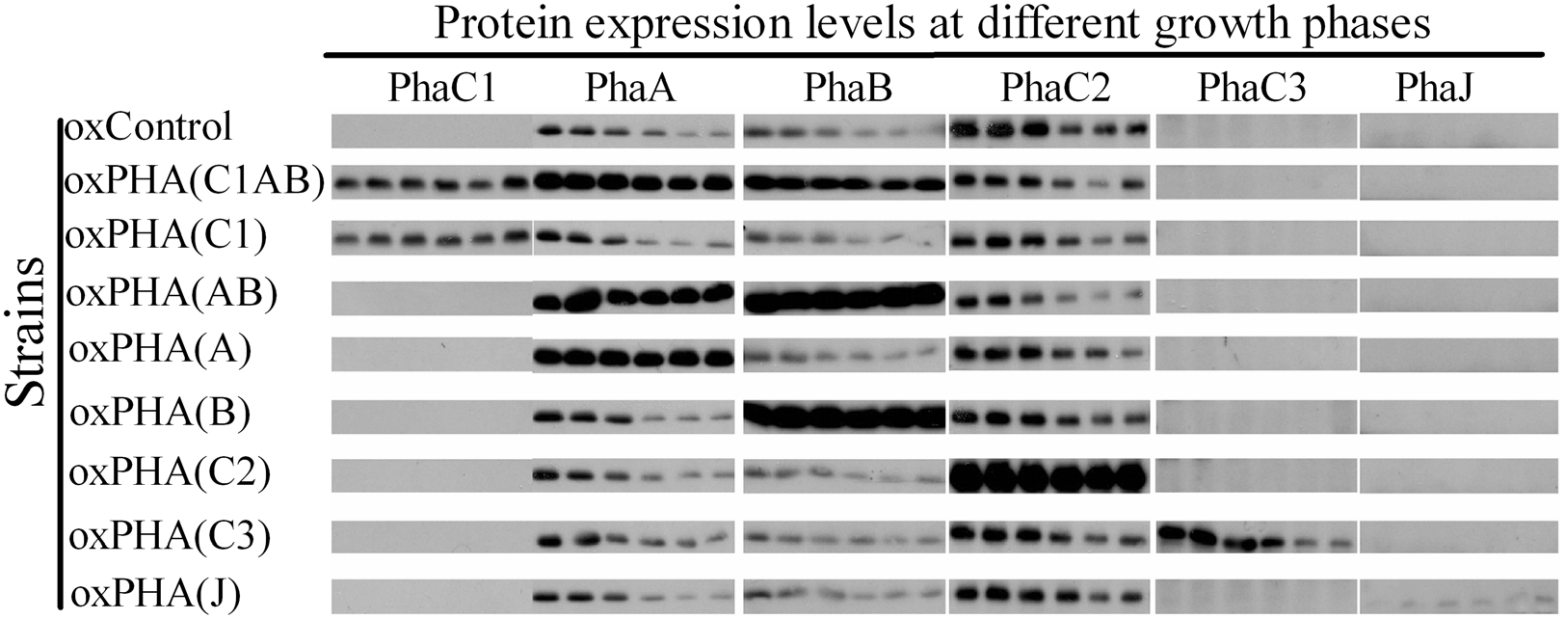
Western blot analysis of PHA biosynthetic proteins in extracts of *R. rubrum* strains after 54, 66, 84, 98, 120, 146-hour cultivation, respectively. For PhaA, PhaB and PhaC2, each lane contained 5 µg of total protein, and for PhaC1, PhaC3 and PhaJ, each lane contained 50 µg of total protein.

These strains (along with the negative control strain generated with the plasmid pUX19-*P_CooF_*, which does not carry any PHA gene sequences) were cultivated anaerobically in a CO_2_/carbon monoxide atmosphere with nitrogen-limiting RRNCO medium containing acetate as a carbon-source. For each strain we determined its growth by monitoring A_680_ (Fig. 3A, 3C and 3E), the accumulation of dry weight biomass (data not shown), and in parallel, at the last six time points of the culture, we determined PHA accumulation by GC-MS analyses (Fig. 3B, 3D and 3F), and the accumulation of each PHA biosynthetic protein via western blot analyses (Fig. 4).

These analyses show that for each recombinant strain we achieved the overexpression of the targeted protein (Fig. 4); for example in the strains oxPHA(C1) and oxPHA(C1AB), PhaC1-expression is at least 50-fold higher than in the oxControl strain. Similarly, the expression of the other targeted PHA biosynthetic enzymes was induced by a factor of between 5- and 50-fold, either individually or in combination. Most significantly for the purpose of interpreting the effect of these genetic manipulations on PHA content, each manipulation only affected the expression of the targeted PHA biosynthetic enzyme and did not significantly affect the expression of the other PHA biosynthetic proteins that were assayed.

The induction of the PHA biosynthetic enzymes resulted in increased PHA levels, which peaked at 66 h after inoculation in the carbon monoxide inducing medium. The PHA content in the control strain carrying the plasmid pUX19-*P_CooF_* that does not carry any PHA gene sequences peaked at about 12% CDW, which was similar to the level obtained with the same control strain not carrying any plasmid. But in the presence of enhanced expressing of *pha* genes, PHA levels increased up to a maximum of about 30% CDW (Figure 3). This compares with the 38% of PHA previously reported in the fermentation of syngas by *R. rubrum* [13]. Although our genetic manipulations increased PHA content, over-expressing PHA biosynthetic genes did not affect any significant change in the monomer composition of the PHA polymer, which is predominantly poly-hydroxybutyrate, with only 0.3%–1% being 3-hydroxyvalerate.

### The Effect of Individually Over-expressing *PhaC* Paralogs and *PhaJ* on PHA Production

To investigate the potential role of each PHA polymerase isozymes to affect PHA production, three strains that individually over-express *phaC1, phaC2* and *phaC3*, (i.e., oxPHA(C1), oxPHA(C2) and oxPHA(C3), respectively) were investigated regarding PHA content and growth. Western blot analysis of protein extracts from these strains show that PhaC1 and PhaC3 levels were at least 50-fold higher than in the control strain, whereas PhaC2 was overexpressed by 25-fold (Fig. 4). All three of these strains accumulate higher levels of PHA than the control strain, by between 1.25-fold and 1.8-fold. However, the PhaC2 over-expressing strain (oxPHA(C2)), which shows the smallest level of over-expression (25-fold increase), results in highest increase of PHA production (increased 1.8-fold); whereas, the other two over-expressing strains (oxPHA(C1) and oxPHA(C3)), increase PHA production by 1.4- and 1.25-fold, respectively (Fig. 3B).

These *in vivo* alterations of PHA production by altering the expression of *phaC* paralogs have a differential effect on the growth of the cultures. Namely, the PhaC2 overexpressing strain, which accumulates more PHA than the other two PhaC-overexpressing strains grew at a significantly faster rate than the control strain, whereas the other two strains demonstrate growth rates similar the control strain (Fig. 3A).

Similar studies were conducted to assess whether altering the expression of the *phaJ* gene would affect the production of PHA. The *phaJ* gene encodes the enzyme 2-enoyl-CoA hydratase, which catalyzes a reaction that is thought to metabolically link intermediates of fatty acid catabolism to intermediates of PHA biosynthesis [26]. Even though PhaJ protein was over-expressed by a factor of at least 5-fold, this modification had relatively small effect on PHA production (increased 1.3-fold), and did not significantly affect the initial growth rate of the strain (Fig. 3C and 3D). Presupposing that the phaJ protein is involved in metabolically connecting fatty acid catabolism with PHA biosynthesis, the finding that *phaJ* overexpression does not affect PHA accumulation is consistent with the probable low rate of fatty acid catabolism when *R. rubrum* is grown on the acetate-carbon source.

### The Effect of Over-expressing Individually or in Combination the PHA-operon Located Genes on PHA Production

The *R. rubrum* genome contains a single three-gene operon (*phaC1-phaA-phaB*) that is recognizable as the PHA biosynthetic operon based upon sequence homology with other orthologs from many microbial genomes. The effect of individually over-expressing each of these *PHA* operon-located biosynthetic genes on PHA accumulation was investigated by using the strains oxPHA(C1), oxPHA(A), and oxPHA(B). In addition, using the strains resulting from recombination at each of the *PHA* operon-located genes we investigated the effect of overexpressing a combination of these genes, i.e., oxPHA(AB) and oxPHA(C1AB) (Table 1). Western blot analysis of extracts from strain oxPHA(A), oxPHA(B) and oxPHA(C1) shows that the expression level of PhaA, PhaB, PhaC1 proteins increased by 5-, 50- and >50-fold, over the control strain, respectively (Fig. 4). In the strain oxPHA(AB), PhaA and PhaB levels are 5- and 50-fold higher than in the control strains, and in the strain oxPHA(ABC1), PhaA, PhaB and PhaC1 levels are increased 5-, 7- and >50-fold, respectively.

All five recombinant strains produce significantly more PHA than the control strain (Fig. 3F). However, the strain oxPHA(B) and oxPHA(AB) expresses the highest level of PHA production (increased by 2.2-fold); the other three recombinant strains (oxPHA(C1), oxPHA(A) and oxPHA(C1AB)), increased PHA levels by about 1.4-, 1.4- and 1.7-fold, respectively (Fig. 3F). These results indicate that the expression level of *phaB* is a major limiting determinant of PHA production.

This conclusion is based on the following comparisons. First, overexpressing *phaB* by 50-fold (either individually or in combination with *phaA*) provides the maximal increase in PHA content (from 13% to 28% and 29% of CDW, respectively). Hence, there is little additive effect in co-overexpressing both *phaB* and *phaA* on PHA levels over individually overexpressing *phaB*. Second, when *phaC1* overexpression is added to this strain (i.e., strain oxPHA(C1AB)), PHA level is at 22% of CDW, and this is due primarily to the fact that *phaB* expression level is only 7-fold higher than the control strain. Finally, when *phaC1* or *phaA* is individually overexpressed, it has the minimal effect on enhancing PHA levels.

### Growth Rates of PHA Over-accumulating Strains

**Figure 5:**
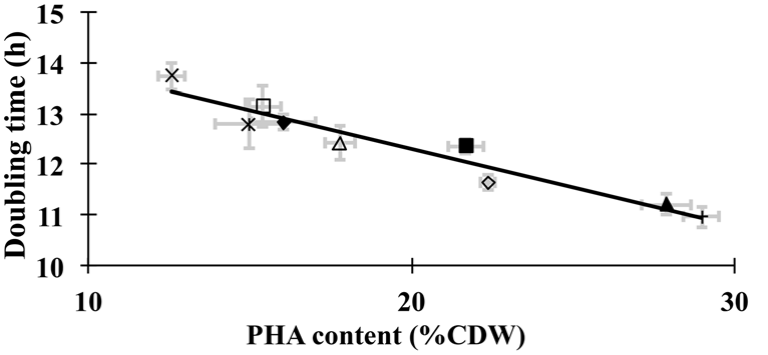
Correlation between culture doubling time and the maximum PHA content of *R. rubrum* strains. *R. rubrum* strains were: oxControl (**×**), oxPHA(C1) (**□**), oxPHA(C2) (**◊**), oxPHA(C3) (*), oxPHA(A) (**△**), oxPHA(B) (**▴**),oxPHA(AB) (**+**), oxPHA(C1AB) (**▪**), oxPHA(J) (**♦**). Data represent averages from triplicate biological samples, and error bars indicate the standard error.

The results indicate that the growth rates of cultures (measured as the initial doubling time) are correlated with the maximal PHA accumulating capacity of the strain (Fig. 5). Specifically, the wild-type control strain that accumulates the lowest levels of PHA, showed the slowest growth rate (longest doubling time), and all recombinant strains, which accumulate higher levels of PHA show increased growth rates. Among all the strains evaluated, the strains oxPHA(AB) and oxPHA(B), accumulate the highest levels of PHA (about 30% of CDW), and they show the fastest growth rates. Figure 5 illustrates that in fact there’s a linear relationship between PHA levels and the initial growth rates of the cultures. The data therefore imply that PHA accumulation contributes to *R. rubrum* growth under anaerobic conditions, in nitrogen-limiting RRNCO medium.

## Discussion

The fermentation-based chemical reduction of biomass carbon, via thermolytic deconstruction of the biomass through a syngas intermediate has been previously explored [12], [13]. Elucidating the metabolic controlling mechanisms that influence the production of PHA by *R. rubrum* cells grown in anaerobic conditions, on chemically simple carbon precursors should further facilitate the use of this organism as a model for syngas fermentation to produce valuable bio-products. As a model, in this study we evaluated the effect of over-expressing *R. rubrum*-encoded PHA biosynthetic genes on PHA production and cellular growth. The strains investigated in these experiments explored the metabolic regulatory points of PHA biosynthesis when *R. rubrum was* grown on nitrogen-limiting medium, with a carbon source composed of CO_2_, CO and acetate. Our results show that *R. rubrum* strains can accumulate up to 30% PHA of CDW, which is composed of 99.4% polyhydroxybutyrate and 0.6% polyhydroxyvalerate. These evaluations demonstrate the potential of using *R. rubrum* for producing a value-added bio-based product from chemically simple carbon sources. Our bioengineering experiments addressed three questions: 1) which of the three *phaC* paralogs in the *R. rubrum* genome is most significant in PHA production? 2) Which of the enzymes in the three-reaction pathway from acetyl-CoA to PHA is limiting PHA production? And 3) Is *phaJ* limiting PHA production in syngas fermentation?

The data presented herein establish that over-expressing *phaC2* results in increased production of PHA, while the impact of over-expressing *phaC1* is less, and over-expressing *phaC3* has little, to no effect. The conclusion that of the three *phaC* paralogs, *phaC2* is the most significant in determining PHA accumulation, is based on the characterization of individual over-expressing alleles, and is corollary to our prior characterizations of individual *phaC* deletion-mutants, which also indicated that the *PhaC2* paralog is the major polymerase isoform for producing PHA [16]. Although prior studies with *Aeromonas hydrophila, Aeromonas caviae* [27], [28], and *Pseudomonas oleovorans* [29] have examined the importance of over-expressing *phaC* on increasing PHA production, the genomes of, each of these organisms harbor only a single-copy of the *phaC* gene, which are located within the *PHA* biosynthetic operon. Moreover, whereas the former two studies enhanced PHA productivity by overexpressing the polymerase, the latter did not significantly increase PHA production under nitrogen limiting conditions.

Characterizations of the three genes that constitute the *PHA* biosynthetic operon establish that *phaB* is the key genetic determinant of PHA productivity. This is consistent with the prior kinetic study of PHA biosynthesis from pentanoic acid by *Alcaigenes eutrophus* [30], but contrasts with the studies that suggest that PhaA is the key enzyme determining PHA production [31], [32]. *PhaB* encodes the NADPH-dependent enzyme that reduces acetoacetyl-CoA to 3-hydrobutyryl-CoA. Therefore, there is a thermodynamic rationale for the energy requiring reaction to be limiting the generation of PHA, a product whose major physiological role is to store reduced carbon as a means of storing energy.

Over-expressing *phaJ* had little effect on PHA production, in these conditions. This is partly predictable based on prior characterization of this gene in *Aeromonas hydrophila* and *Aeromonas caviae* [27], [28], and the fact that the reaction catalyzed by PhaJ, links fatty acid catabolism and PHA biosynthesis. Specifically, it’s energetically unfeasible of an organism to set up a futile cycle between fatty acid biosynthesis (from CO_2_, CO and acetate as carbon sources) and cycle the resulting fatty acids through catabolism in order to produce PHA from the intermediates of fatty acid catabolism.

Additional effectors that have to be considered to enhance the productivity of PHA are processes that stabilize the polymer, which includes eliminating or reducing the rate of degradation. The PHA polymer is found in “organelle” like structures or granules, sometimes called “carbonosomes” [33], and these are considered to be analogous to oil droplets or oleosomes, which occur in eukaryotic organisms [34]. As with oleosomes that are stabilized by a coating of a phospholipid monolayer and the protein oleosin [35], carbonosomes are coated with a similar lipid monolayer and the protein, phasin encoded by the *phaP* gene [33]. Indeed, there are experimental outcomes that indicate a regulatory role for phasin [36] and oleosin [37] in determining the levels of PHA and oil accumulation, respectively.

The other metabolic function that can affect PHA levels in cells is the rate of polymer turnover, which is catalyzed by PHA depolymerase, encoded by *phaZ* genes [33]. These are difficult enzymes to assay because of the polymeric nature of their substrates, but evidence suggests that they are associated with the carbonosome granules, and cells express a set of paralogs that display different substrate specificities [38].

Finally, our results indicate that PHA content enhances growth rate of *R. rubrum* (Fig. 5). Though the underlying mechanism of this phenomenon is still unclear, previous studies have shown that PHA enhances resistance to stresses (radiation, desiccation and osmotic pressure) [39], and this resistance may be mediated by increasing of the levels of the growth regulator guanosine-tetraphosphate (ppGpp), which enhances PHA degradation during the stress of *Pseudomonas oleovorans* [40].

## Supporting Information

File S1 This file contains: Tables S1–S4.
(DOC)

## References

[1] Kunkes EL, Simonetti DA, West RM, Serrano-Ruiz JC, Gartner CA, et al (2008) Catalytic conversion of biomass to monofunctional hydrocarbons and targeted liquid-fuel classes. Science 322: 417–421. 18801970 10.1126/science.1159210

[2] Nikolau BJ, Perera MA, Brachova L, Shanks B (2008) Platform biochemicals for a biorenewable chemical industry. The Plant journal: for cell and molecular biology 54: 536–545. 18476861 10.1111/j.1365-313X.2008.03484.x

[3] Guéhenneux G, Baussand P, Brothier M, Poletiko C, Boissonnet G (2005) Energy production from biomass pyrolysis: a new coefficient of pyrolytic valorisation. Fuel 84: 773–739.

[4] Jarboe LR, Wen Z, Choi D, Brown RC (2011) Hybrid thermochemical processing: fermentation of pyrolysis-derived bio-oil. Appl Microbiol Biotechnol 91: 1519–1523. 21789490 10.1007/s00253-011-3495-9

[5] Kerby RL, Ludden PW, Roberts GP (1995) Carbon monoxide-dependent growth of Rhodospirillum rubrum. J Bacteriol 177: 2241–2244. 7721719 10.1128/jb.177.8.2241-2244.1995 PMC176875

[6] Pfennig N, Trpüer HG (1978) The Rhodospirillales (phototrophic or photosynthetic bacteria). In: R. K Clayton and W. R Sistrom, editors. Taxonomy of the Rhodospirillales. New York: Plenum Press. 17–27.

[7] Steinbüchel A, Hein S (2001) Biochemical and Molecular Basis of Microbial Synthesis of Polyhydroxyalkanoates in Microorganisms Adv Biochem Eng Biotechnol. 71: 81–123. 10.1007/3-540-40021-4_3 11217418

[8] Kim YB, Lenz RW (2001) Polyesters from microorganisms. Adv Biochem Eng Biotechnol 71: 51–79. 11217417 10.1007/3-540-40021-4_2

[9] Madison LL, Huisman GW (1999) Metabolic engineering of poly(3-hydroxyalkanoates): from DNA to plastic. Microbiol Mol Biol Rev 63: 21–53. 10066830 10.1128/mmbr.63.1.21-53.1999 PMC98956

[10] Steinbüchel A (2001) Perspectives for Biotechnological Production and Utilization of Biopolymers: Metabolic Engineering of Polyhydroxyalkanoate Biosynthesis Pathways as a Successful Example. Macromol Biosci 1: 1–24.

[11] Brandl H, Knee EJ Jr, Fuller RC, Gross RA, Lenz RW (1989) Ability of the phototrophic bacterium Rhodospirillum rubrum to produce various poly (beta-hydroxyalkanoates): potential sources for biodegradable polyesters. Int J Biol Macromol 11: 49–55. 2518731 10.1016/0141-8130(89)90040-8

[12] Choi D, Chipman DC, Bents SC, Brown RC (2010) A techno-economic analysis of polyhydroxyalkanoate and hydrogen production from syngas fermentation of gasified biomass. Applied biochemistry and biotechnology 160: 1032–1046. 19247588 10.1007/s12010-009-8560-9

[13] Do YS, Smeenk J, Broer KM, Kisting CJ, Brown R, et al (2007) Growth of Rhodospirillum rubrum on synthesis gas: conversion of CO to H2 and poly-beta-hydroxyalkanoate. Biotechnol Bioeng 97: 279–286. 17054121 10.1002/bit.21226

[14] Henstra AM, Sipma J, Rinzema A, Stams AJ (2007) Microbiology of synthesis gas fermentation for biofuel production. Curr Opin Biotechnol 18: 200–206. 17399976 10.1016/j.copbio.2007.03.008

[15] Reiser SE, Mitsky TA, Gruys KJ (2000) Characterization and cloning of an (R)-specific trans-2,3-enoylacyl-CoA hydratase from Rhodospirillum rubrum and use of this enzyme for PHA production in Escherichia coli. Appl Microbiol Biotechnol 53: 209–218. 10709984 10.1007/s002530050010

[16] Jin H, Nikolau BJ (2012) The role of genetic redundancy in Polyhydroxyalkanoate Polymerases in PHA biosynthesis in *Rhodospirillum rubrum*. J Bacteriol doi:10.1128/JB.01111-12. 10.1128/JB.01111-12 PMC3458679 22865850

[17] Hustede E, Steinbuchel A, Schlegel HG (1992) Cloning of poly(3-hydroxybutyric acid) synthase genes of Rhodobacter sphaeroides and Rhodospirillum rubrum and heterologous expression in Alcaligenes eutrophus. FEMS Microbiol Lett 72: 285–290. 1499989 10.1016/0378-1097(92)90476-5

[18] Clemente T, Shah D, Tran M, Stark D, Padgette S, et al (2000) Sequence of PHA synthase gene from two strains of Rhodospirillum rubrum and in vivo substrate specificity of four PHA synthases across two heterologous expression systems. Appl Microbiol Biotechnol 53: 420–429. 10803898 10.1007/s002530051636

[19] Kerby RL, Hong SS, Ensign SA, Coppoc LJ, Ludden PW, et al (1992) Genetic and physiological characterization of the Rhodospirillum rubrum carbon monoxide dehydrogenase system. J Bacteriol 174: 5284–5294. 1644755 10.1128/jb.174.16.5284-5294.1992 PMC206364

[20] Brandl H, Gross RA, Lenz RW, Fuller RC (1988) Pseudomonas oleovorans as a Source of Poly(beta-Hydroxyalkanoates) for Potential Applications as Biodegradable Polyesters. Appl Environ Microbiol 54: 1977–1982. 16347708 10.1128/aem.54.8.1977-1982.1988 PMC202789

[21] Sambrook J, Russell DW (2001) Molecular Cloning: A laboratory manual. Cold Spring Harbor, New York: Cold Spring Harbor Laboratory Press.

[22] He Y, Gaal T, Karls R, Donohue TJ, Gourse RL, et al (1999) Transcription activation by CooA, the CO-sensing factor from Rhodospirillum rubrum. The interaction between CooA and the C-terminal domain of the alpha subunit of RNA polymerase. J Biol Chem 274: 10840–10845. 10196160 10.1074/jbc.274.16.10840

[23] Zhang Y, Pohlmann EL, Ludden PW, Roberts GP (2001) Functional characterization of three GlnB homologs in the photosynthetic bacterium Rhodospirillum rubrum: roles in sensing ammonium and energy status. J Bacteriol 183: 6159–6168. 11591658 10.1128/JB.183.21.6159-6168.2001 PMC100091

[24] Liang JH, Nielsen GM, Lies DP, Burris RH, Roberts GP, et al (1991) Mutations in the draT and draG genes of Rhodospirillum rubrum result in loss of regulation of nitrogenase by reversible ADP-ribosylation. J Bacteriol 173: 6903–6909. 1938894 10.1128/jb.173.21.6903-6909.1991 PMC209044

[25] Aich S, Delbaere LT, Chen R (2001) Continuous spectrophotometric assay for beta-glucuronidase. Biotechniques 30: 846–850. 11314267 10.2144/01304rr02

[26] Fukui T, Shiomi N, Doi Y (1998) Expression and characterization of (R)-specific enoyl coenzyme A hydratase involved in polyhydroxyalkanoate biosynthesis by Aeromonas caviae. J Bacteriol 180: 667–673. 9457873 10.1128/jb.180.3.667-673.1998 PMC106937

[27] Han J, Qiu YZ, Liu DC, Chen GQ (2004) Engineered Aeromonas hydrophila for enhanced production of poly(3-hydroxybutyrate-co-3-hydroxyhexanoate) with alterable monomers composition. FEMS Microbiol Lett 239: 195–201. 15451119 10.1016/j.femsle.2004.08.044

[28] Fukui T, Kichise T, Iwata T, Doi Y (2001) Characterization of 13 kDa granule-associated protein in Aeromonas caviae and biosynthesis of polyhydroxyalkanoates with altered molar composition by recombinant bacteria. Biomacromolecules 2: 148–153. 11749166 10.1021/bm0056052

[29] Kraak MN, Smits TH, Kessler B, Witholt B (1997) Polymerase C1 levels and poly(R-3-hydroxyalkanoate) synthesis in wild-type and recombinant Pseudomonas strains. J Bacteriol 179: 4985–4991. 9260937 10.1128/jb.179.16.4985-4991.1997 PMC179353

[30] Doi Y, Kawaguchi Y, Koyama N, Nakamura S, Hiramitsu M, et al (1992) Synthesis and degradation of polyhydroxyalkanoates in Alcaligenes eutrophus. FEMS Microbiol Lett 103: 103–108.

[31] Senior PJ, Dawes EA (1971) Poly-β-hydroxybutyrate biosynthesis and the regulation of glucose metabolism in Azotobacter beijerinckii. Biochem J 125: 55–66. 4400642 10.1042/bj1250055 PMC1178025

[32] Oeding V, Schlegel HG (1973) Beta-ketothiolase from Hydrogenomonas eutropha H16 and its significance in the regulation of poly-beta-hydroxybutyrate metabolism. Biochem J 134: 239–248. 4198758 10.1042/bj1340239 PMC1177804

[33] Jendrossek D (2009) Polyhydroxyalkanoate granules are complex subcellular organelles (carbonosomes). J Bacteriol 191: 3195–3202. 19270094 10.1128/JB.01723-08 PMC2687172

[34] Purkrtova Z, Jolivet P, Miquel M, Chardot T (2008) Structure and function of seed lipid-body-associated proteins. C R Biol 331: 746–754. 18926488 10.1016/j.crvi.2008.07.016

[35] Maurer S, Waschatko G, Schach D, Zielbauer BI, Dahl J, et al (2013) The role of intact oleosin for stabilization and function of oleosomes. The journal of physical chemistry B 117: 13872–13883. 24088014 10.1021/jp403893n

[36] Potter M, Madkour MH, Mayer F, Steinbuchel A (2002) Regulation of phasin expression and polyhydroxyalkanoate (PHA) granule formation in Ralstonia eutropha H16. Microbiology 148: 2413–2426. 12177335 10.1099/00221287-148-8-2413

[37] Kim HU, Jung SJ, Lee KR, Kim EH, Lee SM, et al (2013) Ectopic overexpression of castor bean LEAFY COTYLEDON2 (LEC2) in Arabidopsis triggers the expression of genes that encode regulators of seed maturation and oil body proteins in vegetative tissues. FEBS open bio 4: 25–32. 10.1016/j.fob.2013.11.003 PMC3863707 24363987

[38] Brigham CJ, Reimer EN, Rha C, Sinskey AJ (2012) Examination of PHB Depolymerases in Ralstonia eutropha: Further Elucidation of the Roles of Enzymes in PHB Homeostasis. AMB Express 2: 26. 22537946 10.1186/2191-0855-2-26 PMC3430594

[39] Tal S, Okon Y (1985) Production of the reserve material poly-β-hydroxybutyrate and its function in Azospirillum brasilense Cd. Can J Microbiol 31: 608–613.

[40] Ruiz JA, Lopez NI, Fernandez RO, Mendez BS (2001) Polyhydroxyalkanoate degradation is associated with nucleotide accumulation and enhances stress resistance and survival of Pseudomonas oleovorans in natural water microcosms. Appl Environ Microbiol 67: 225–230. 11133449 10.1128/AEM.67.1.225-230.2001 PMC92552

