# Supplementary material for: Evaluating PHA Productivity of Bioengineered Rhodosprillum rubrum
Source: PLoS One. 2014 May 19;9(5):e96621. doi: 10.1371/journal.pone.0096621 (PMC4026134; doi:10.1371/journal.pone.0096621)
Supplement: File S1 — This file contains: Tables S1–S4. (DOC) [file pone.0096621.s001.doc]

**Evaluating PHA productivity of bioengineered *Rhodosprillum rubrum***

Huanan Jin^1^ and Basil J. Nikolau^1,2^

Department of Biochemistry, Biophysics and Molecular Biology^1^, and Center for Biorenewable Chemicals^2^, Iowa State University, Ames, Iowa 50011

**Supplemental materials**

Table S1. Bacterial strains, and plasmids used in this study.

| Strains, plasmid or oligonucleotides | Relevant characteristic(s) | Source or reference |
| --- | --- | --- |
| **strains** |  |  |
| *R. rubrum* ATCC 11170 | Source of PHA | ATCC |
| *E. coli* DH5α | Cloning strain | Invitrogen |
| *E. coli* S17-1 | Mobilizing strain | (Simon et al. 1983) |
| *E. coli* BL21-AI | Expression strain | Invitrogen |
| **plasmids** |  |  |
| pPCR2.1 | TA Cloning vector | Invitrogen |
| pENTR/SD/D-TOPO | Gateway cloning vector | Invitrogen |
| pDEST15 | Expression vector with GST tag | Invitrogen |
| pDEST17 | Expression vector with His tag | Invitrogen |
| pUX19 | Suicide vector for *R. rubrum* | (Zhang et al. 2001) |
| pUX19-PT26 | pUX19::P_CooF_ -T_CooF_ | This study |
| pUX19-PT26-phaC1 | pUX19::P_CooF_ -*phaC*-T_CooF_ | This study |
| pUX19-PT26-phaC2 | pUX19::P_CooF_ –*phaC2*-T_CooF_ | This study |
| pUX19-PT26-phaC3 | pUX19::P_CooF_ –*phaC3*-T_CooF_ | This study |
| pUX19-PT26-phaJ | pUX19::P_CooF_ -*phaJ*-T_CooF_ | This study |
| pUX19-PT26-phaA | pUX19::P_CooF_ -*phaA*-T_CooF_ | This study |
| pUX19-PT26-phaB | pUX19::P_CooF_ -*phaB*-T_CooF_ | This study |

Table S2. Oligonucleotides used in this study^a^

| Fragment amplified | Primer name | Primer sequence |
| --- | --- | --- |
| *CooFSCTJ* promoter | PUS-*XbaI* | *TCTAGA*TGGCCCTGGGCGAGCAC |
|  | PDS- *NdeI* | *CATATG*TACTGCCCTCCACGCATTGA |
| *CooFSCTJ* terminator | TUS-*SacI* | *GAGCTC*TGGCGCGGATGATCTCTTGT |
|  | TDS- *XhoI* | *CTCGAG*TGCTATAAAACCGATGAATCTC |
| *phaJ* | phaJUS-*NdeI* | *CATATG*AGCGCCGACGACCTGAT |
|  | phaJDS- *sacI* | *GAGCTC*TTAGCCGCGCGCGGGAACCA |
| *phaC* | phaC1US-*SacI* | *CATATG*ACCCAGCCGCCGCCCCC |
|  | phaC1DS- *XhoI* | *GAGCTC*CTATCGTCCCGCCAGACC |
| *phaA* | phaAUS-*SacI* | *CATATG*ACCGATATCGTCATTGC |
|  | phaADS- *XhoI* | *GAGCTC*TTAGCGCTCGACGCAGAGCG |
| *phaB* | phaBUS-*SacI* | *CATATG*ACGAAAGGGCGTGTCGC |
|  | phaBDS- *XhoI* | *GAGCTC*TTAATACATGTGCTGGCCGC |
| *phaC2* | phaC2US-*SacI* | *CATATG*GCCAATCAGGGCAGCGA |
|  | phaC2DS- *XhoI* | *GAGCTC*CTAGCCGGCGCGCACCTTCA |
| *phaC3* | phaC3US-*SacI* | *CATATG*ACCGACACGCGCGCCGA |
|  | phaC3DS- *XhoI* | *GAGCTC*TCAGATCCTAACCTTGACGTAAG |

^a^the italized sequences indicate the restriction sites.

Table S3. SMN Medium ^a^ (per liter) (Kerby et al. 1992)

| Component |  |
| --- | --- |
| MOPS (morpholinopropanesulfonic acid) | 10.5 g |
| Malic acid | 4 g |
| NH_4_Cl | 1 g |
| H_3_BO_4_ | 2.8 mg |
| Disodium EDTA | 20 mg |
| Ferric citrate | 4 mg |
| Na_2_MoO_4_ | 1 mg |
| KH_2_PO_4_ | 600 mg |
| K_2_HPO_4_ | 900 mg |
| MgSO_4_ | 250 mg |
| CaCl_2_ | 100 mg |
| Biotin | 1 μg |
| Casein enzyme hydrolysate | 3 g |
| Yeast extract | 3 g |
| NiCl_2_ | 1.3 mg |

^a^Adjust pH to 7.0 with NaOH solution.

Table S4. RRNCO Medium^a^ (per liter) (ammonium chloride omitted) (Kerby et al. 1995)

| Medium component |  |
| --- | --- |
| Biotin | 2 μg |
| H_3_BO_4_ | 2.8 mg |
| Disodium EDTA | 20 mg |
| Ferric citrate | 4 mg |
| Na_2_MoO_4_ | 1 mg |
| MgSO_4_•7H_2_O | 250 mg |
| CaCl_2_•2H_2_O | 132 mg |
| NiCl_2_ | 2.6 mg |
| Yeast extract | 1 g |
| MOPS (morpholinopropanesulfonic acid) | 2.1 g |
| CH_3_COONa | 0.82 g |
| [K](http://en.wikipedia.org/wiki/Potassium)[H](http://en.wikipedia.org/wiki/Hydrogen)_2_[P](http://en.wikipedia.org/wiki/Phosphorus)[O](http://en.wikipedia.org/wiki/Oxygen)_4_ | 1.3 g |
| Na_2_S•9H_2_O | 100 mg |
| NaHCO_3_ | 1.05 g |

^a^Adjust pH to 7.1 with NaOH solution and filter-sterilized CO was added prior to inoculation.

**References**

Kerby RL, Hong SS, Ensign SA, Coppoc LJ, Ludden PW, Roberts GP (1992) Genetic and physiological characterization of the *Rhodospirillum rubrum* carbon monoxide dehydrogenase system. J Bacteriol 174(16):5284-94

Kerby RL, Ludden PW, Roberts GP (1995) Carbon monoxide-dependent growth of *Rhodospirillum rubrum*. J Bacteriol 177(8):2241-4

Simon R, Priefer U, Pühler A (1983) A Broad Host Range Mobilization System for *In Vivo* Genetic Engineering: Transposon Mutagenesis in Gram Negative Bacteria. Nat Biotechnol 1:784 - 791

Zhang Y, Pohlmann EL, Ludden PW, Roberts GP (2001) Functional characterization of three GlnB homologs in the photosynthetic bacterium *Rhodospirillum rubrum*: roles in sensing ammonium and energy status. J Bacteriol 183(21):6159-68
